# Four-Point Computed Tomography Scores for Evaluation of Occult Peritoneal Metastasis in Patients with Gastric Cancer: A Region-to-Region Comparison with Staging Laparoscopy

**DOI:** 10.1245/s10434-019-07812-y

**Published:** 2020-01-21

**Authors:** Zi-Yu Li, Lei Tang, Zhe-Min Li, Yan-Ling Li, Jia Fu, Yan Zhang, Xiao-Ting Li, Xiang-Ji Ying, Jia-Fu Ji

**Affiliations:** 1 Gastrointestinal Cancer Center, Peking University Cancer Hospital and Institute, Key Laboratory of Carcinogenesis and Translational Research (Ministry of Education), Beijing, China; 2 Department of Radiology, Peking University Cancer Hospital and Institute, Key Laboratory of Carcinogenesis and Translational Research (Ministry of Education), Beijing, China

## Abstract

**Background:**

Preoperative diagnosis of peritoneal metastasis with gastric cancer remains challenging. This study explored the abnormal computed tomography (CT) signs of occult peritoneal metastasis (OPM) and evaluated it by region-to-region comparison using staging laparoscopy, from which a 4-point CT score system was developed.

**Methods:**

Patients with advanced gastric cancer (stage cT ≥ 2M0) diagnosed by CT were enrolled in the study. Occult peritoneal metastasis detected during staging laparoscopy was compared with preoperative CT to investigate the presence of abnormal signs by a region-to-region comparison. A 4-point CT score system was developed to define the radiologic characteristics. Subsequently, the diagnostic efficacy of the CT score system was prospectively verified.

**Results:**

In this study, 57 OPM regions were detected by staging laparoscopy in 33 of the 385 enrolled patients. The greater omentum was the most frequent site of OPM (38.60%, 22/57), which usually exhibited a smudge-like ground-glass opacity (S-GGO) (90.91%, 20/22) with a mean CT score of 2.14. The parietal and perihepatic peritoneum was the second most common site (22.81%, 13/57). A 4-point CT score system was developed based on the results. A cutoff CT score of 2 or higher was associated with a false-negative rate of 2% (2/99). This CT score system had a sensitivity of 87.5% and a specificity of 76.4% for an OPM-positive diagnosis (area under the curve, 0.848). The agreement between two radiologists on the assigned final score was 76.2% (kappa, 0.5).

**Conclusions:**

Patients with OPM mostly exhibited S-GGO on CT, which should be interpreted cautiously. The 4-point CT score system may improve the pretreatment evaluation of occult peritoneal metastasis, and staging laparoscopy might not be necessary for patients with a score lower than 2.

**Electronic supplementary material:**

The online version of this article (10.1245/s10434-019-07812-y) contains supplementary material, which is available to authorized users.

Peritoneal metastasis (PM) occurs in an estimated 55–60% of patients with metastatic gastric cancer, and accurate diagnosis of PM before treatment is of much clinical significance.^1^^,^^2^ Computed tomography (CT) is the most commonly used noninvasive method for detecting PM.^3^^,^^4^ The typical CT signs of PM usually appear in the latter stages of PM, and a CT diagnosis has a high specificity but a low sensitivity (~ 50%).^4^^,^^5^

In a study of 657 gastric cancer patients, the false-negative rate was 31%.^6^ Therefore, we designed this prospective study to investigate whether signs of occult peritoneal metastasis (OPM) are missed by CT by performing a region-to-region comparison of CT with staging laparoscopy. Furthermore, we verified the potential of a 4-point CT score system for evaluation of OPM.

## Materials and methods

### Patients

This prospective study was performed using an institutional review board-approved protocol, and written informed consent was waived. The inclusion criteria for the study specified advanced gastric cancer (≥ cT2) as assessed by CT, no evidence of metastasis, no previous abdominal surgery, and no previous abdominal malignancies or inflammatory diseases.

The exclusion criteria ruled out typical PM signs diagnosed by CT (diffuse omental nodules or omental cake, severe ascites, obvious irregular thickening with high enhancement of the peritoneum), an interval between CT and laparoscopy longer than 2 weeks, and CT artifacts that prevented observation of peritoneal lesions.

### CT Examination

The enrolled patients underwent an abdominal CT (Discovery CT750 HD scanner; GE Medical Systems, Milwaukee, WI, USA) examination after overnight fasting. Anisodamine and effervescent granules were used to reduce gastrointestinal motility and distend the stomach.

All the patients underwent unenhanced and two-phase dynamic enhanced CT examinations (arterial phase 40 s after contrast media injection; venous phase 70 s after injection). The imaging parameters of the CT examinations were as follows: spectral imaging mode with fast tube voltage switching between 80 and 140 kVp during a single rotation, tube current of 640 mA (the data acquisition time of any given detection view was automatically optimized to provide similar signal strength for both voltages), collimation thickness of 5 mm, reconstruction thickness of 0.625 mm, rotation speed of 0.6 s, helical pitch of 0.984, and CT dose index volume of 22.82 mGy.

Computed tomography images were reconstructed using software designed to decompose projection-based images. An adaptive statistical iterative reconstruction (ASIR) algorithm (index, 30%) was applied to suppress image noise and decrease the radiation dose caused by spectral CT.

The diagnosis was performed with a standardized dynamic window-adjustment procedure on Picture Archiving and Communication Systems workstations. First, a narrow window width/level (*W*_w/l_) was used to locate the primary lesions, with the *W*_w/l_ adjusted wide enough to observe the peritoneal fat status. The optimal window for the detection of PM should clearly display mild grainy background noise of the fat tissue. Second, three-plane images (axial, coronal, and sagittal planes) were observed to facilitate the detection of PM. More details are provided in the Supplementary File.

### Conventional CT Signs of Peritoneal Metastasis^4^

The CT appearances of PM include the “omental cake” sign, a large amount of ascites, and obviously irregular thickening or multiple highly enhanced solid nodules in peritoneal areas.

### Staging Laparoscopy Technique

The technique used for staging laparoscopy is described elsewhere.^7^ A 10-mm disposable trocar (observation hole) was inserted into the sub-umbilicus, followed by insertion of a 10-mm trocar into the right upper quadrant and a 5-mm trocar (operating hole) into the left upper quadrant.

Before any manipulation, 250 mL of warm normal saline was infused into the abdominal cavity. Care was taken to avoid direct contact with the primary tumor. At least 100 mL of fluid was aspirated for cytologic examination. Subsequently, systematic inspection of the abdominal cavity was performed clockwise from the right quadrant. A biopsy of any suspicious lesion was performed.

The entire procedure was recorded by a video recorder. Areas affected by metastasis were identified by a combination of intraoperative observation and postoperative video review, and screen shots were obtained for comparison.

### Region-to-Region Comparison of CT with Staging Laparoscopy

The OPM-positive patients with their diagnosis determined by laparoscopic pathology were enrolled in this step. One laparoscopic surgeon and one radiologist performed the comparisons together. The surgeon recorded the exact OPM-positive locations and obtained intraoperative photographs. Subsequently, the surgeon and the radiologist correlated the OPM areas with the CT counterparts one by one, referring to adjacent organs and vascular structures during the fixed region-to-region comparison workflow. Finally, the radiologist defined the CT signs of these areas and assigned scores (0–3) according to the degree of severity (Fig. S1).

### 4-Point Score System Defining the Radiologic Characteristics of OPM

A 4-point score system was assigned to define the radiologic characteristics of the OPM areas observed on CT according to their appearance and degree of severity (Tables 1, 2 and S1):

**Table 1:** Definition of the computed tomography (CT) scoring system

Score	Free peritoneum	Peritoneum covering organs or tissues
0	No abnormal sign	No lines displayed
1 (mild)	Slightly and homogeneously increased fat density appearing as S-GGO	Slight thickened line
2 (moderate)	Heterogeneously increased density with patchy or intensive S-GGO	Obviously thickened line with enhancement
3 (severe)	Heterogeneously and obviously increased density with intensive S-GGO, multiple strands, curls sign, or blurred-margined small nodules	Obviously thickened line with enhancement and tiny nodules or a small amount of ascites

*S-GGO* smudge-like ground-glass opacity

**Table 2:** Distribution of occult peritoneal metastasis (OPM)-positive areas and averaged computed tomography (CT) scores

Locations	No. of regions	Average CT scores
Greater omentum	22	2.14
Parietal peritoneum	13	1.69
Perihepatic peritoneum	13	0.85
Left subdiaphragm area	3	1.33
Transverse mesocolon	2	2.50
Hepatogastric ligament	2	2.50
Falciform ligament	2	2.00

1. OPM of free peritoneum (not attached to organs or tissues)
  Score 0: no abnormal sign
  Score 1 (mild): slightly and homogeneously increased fat density appearing as smudge-like ground-glass opacity (S-GGO)
  Score 2 (moderate): heterogeneously increased density with patchy or intensive S-GGO
  Score 3 (severe): heterogeneously and obviously increased density with intensive S-GGO, multiple strands, curls sign, or blurred-margined small nodules
2. OPM of peritoneum covering organs or tissues
  Score 0: no lines displayed;
  Score 1 (mild): slightly thickened line
  Score 2 (moderate): obviously thickened line with enhancement
  Score 3 (severe): obviously thickened line with enhancement and tiny nodules or a small amount of ascites

### Prospective Validation

The diagnostic accuracy of the defined CT score system was validated through a prospective study involving a cohort of 143 consecutive patients between August 2016 and January 2018. One radiologist assigned the scores to the enrolled patients according to the proposed system mentioned earlier before staging laparoscopy, and the highest score of suspicious areas in the same patient was recorded. To assess the interrater reliability of the score system, another radiologist assigned the scores to the same patients independently.

### Statistical Analyses

Spearman correlation analysis was used to compare the CT scores and staging laparoscopy results for diagnosis of OPM. Receiver operating characteristic (ROC) curve analysis was performed to evaluate the diagnostic performance of the CT score system for predicting OPM and to determine the optimal cutoff values. A statistically significant difference was reported if the *p* value was lower than 0.05 (two-sided). The statistical analyses were performed using statistical software (SPSS for Windows, version 20; SPSS Inc, Chicago IL, USA).

## Results

This study enrolled 472 patients from August 2013 to June 2016. The diagnosis for 55 of these patients was PM positivity by CT examination. Of the remaining 417 patients, 29 were excluded from the study because of intervals longer than 2 weeks between the CT and staging laparoscopy. Three patients were excluded because of severe artifacts on CT that influenced the diagnosis of PM. The remaining 385 patients were included in the study. For 33 of these patients with 57 regions, an OPM-positive condition was diagnosed by staging laparoscopy, and the areas of all the regions could be correspondingly located on CT images (Tables 1, 2 and S1).

**Fig. 1:**
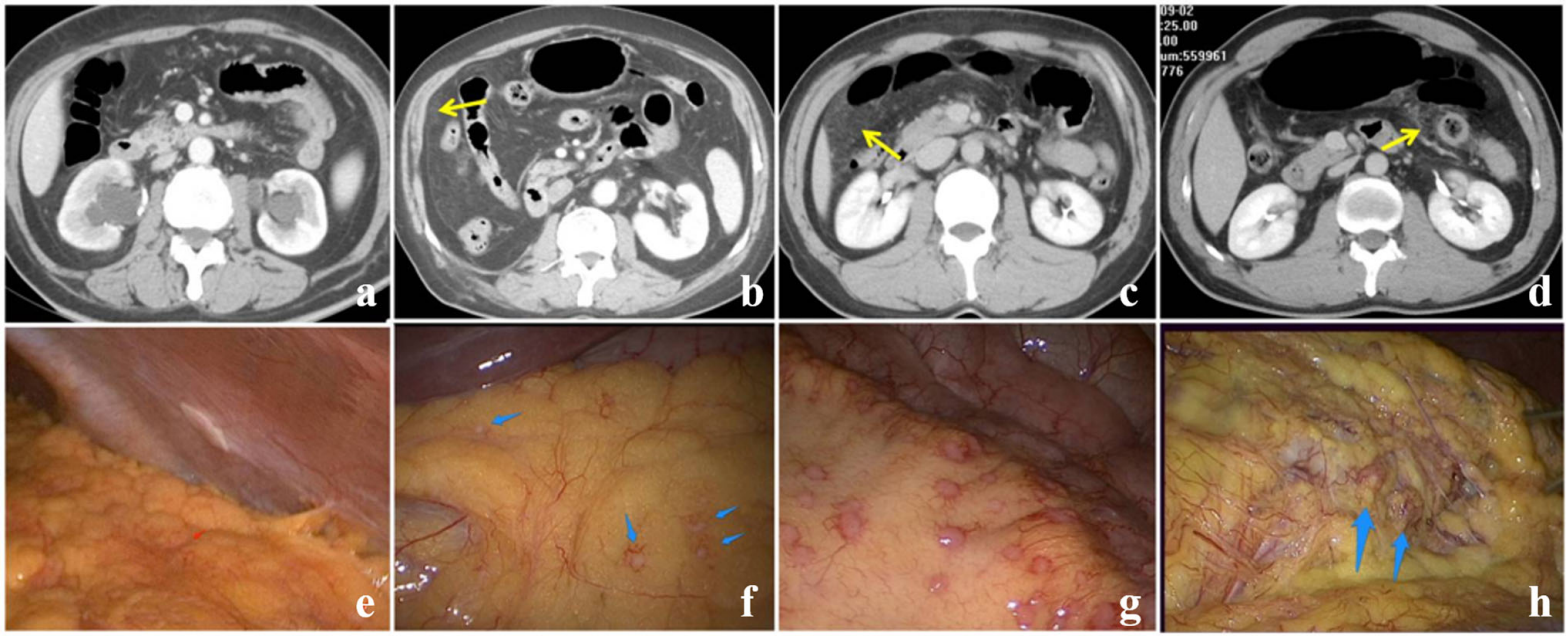
Occult peritoneal metastasis (OPM) score of free peritoneum. **a** OPM grade 0 (no abnormal signs). **b** OPM grade 1 (slightly increased fat density appearing as S-GGO). **c** OPM grade 2 (increased fat density with patchy S-GGO). **d** OPM grade 3 (obviously increased fat density with multiple strands and blurred small nodules). **e**–**h** Intraoperative screen shots of staging laparoscopy corresponding to the aforementioned computed tomography (CT) findings. The degree of metastasis is displayed from light to severe. S-GGO, smudge-like ground-glass opacity

Of the 22 patients who showed involvement of the greater omentum, 2 exhibited no abnormal CT signs (Fig. 1a). All the remaining 20 patients exhibited the S-GGO sign, with 2 patients graded a mild (Fig. 1b), 9 patients graded as moderate (Fig. 1c), and 9 patients graded as severe (Fig. 1d). Laparoscopy detected involvement of the right omentum in 10 patients, the left omentum in 6 patients, the middle omentum in 3 patients, the middle and right omentum in 1 patient, and the left, middle, and right omentum in 2 patients.

**Fig. 2:**
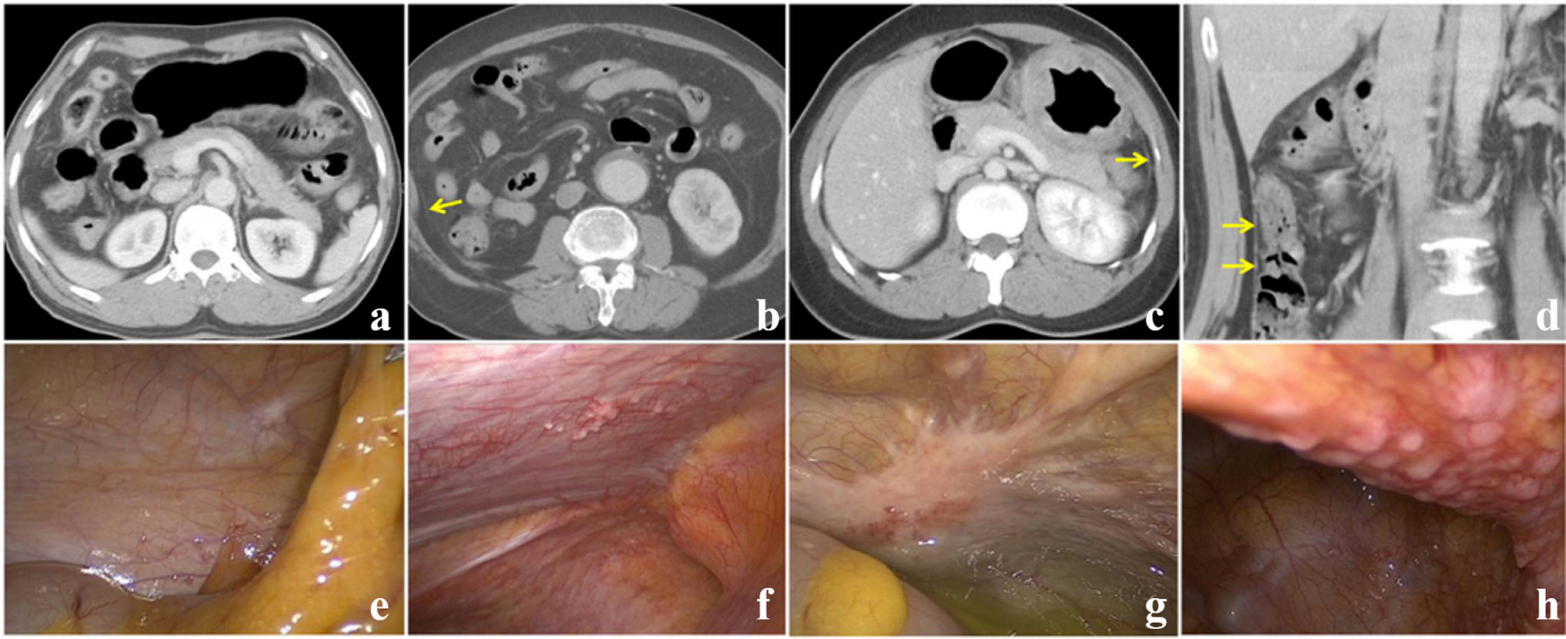
Occult peritoneal metastasis (OPM) score of the peritoneum attached to the organs or tissues. **a** OPM grade 0 (no thickening of the peritoneum observed). **b** OPM grade 1 (slight thickening with a smooth contour). **c** OPM grade 2 (obvious thickening with enhancement). **d** OPM grade 3 (obvious thickening with enhancement and tiny nodules). **e**–**h** Intraoperative screen shots of staging laparoscopy corresponding to the aforementioned computed tomography (CT) findings. The degree of metastasis is displayed from light to severe

The parietal peritoneum was the second most commonly involved region, with 13 regions detected by laparoscopy, 8 on the left side (average CT score, 1.63) and 5 on the right side (average CT score, 1.80). Two patients exhibited no abnormal signs on CT (Fig. 2a), whereas 9 of the remaining 11 patients (81.8%) exhibited peritoneal thickening (Fig. 2b, c). Of these 11 patients, 2 exhibited a small indefinite nodular appearance (Fig. 2d).

The perihepatic peritoneum metastatic regions had the lowest CT score (0.85). Among the 13 regions detected by laparoscopy, 8 showed no abnormal imaging findings (Fig. S2a). Only two cases scored 3, including one with a diagnosis determined indirectly by the existence of perihepatic ascites (Fig. S2b).

The other areas uncommonly affected by OPM were the transverse mesocolon, which exhibited the S-GGO sign with multiple strands and small blurred nodules in the area between the pancreas and the transverse colon (Fig. S3a); the hepatogastric ligament, which exhibited increased fat density with patchy S-GGO in the area between the lesser curvature of the stomach and the hepatic portal (Fig. S3b); and the falciform ligament, which exhibited the S-GGO sign with multiple strands (Fig. S3c).

**Fig. 3:**
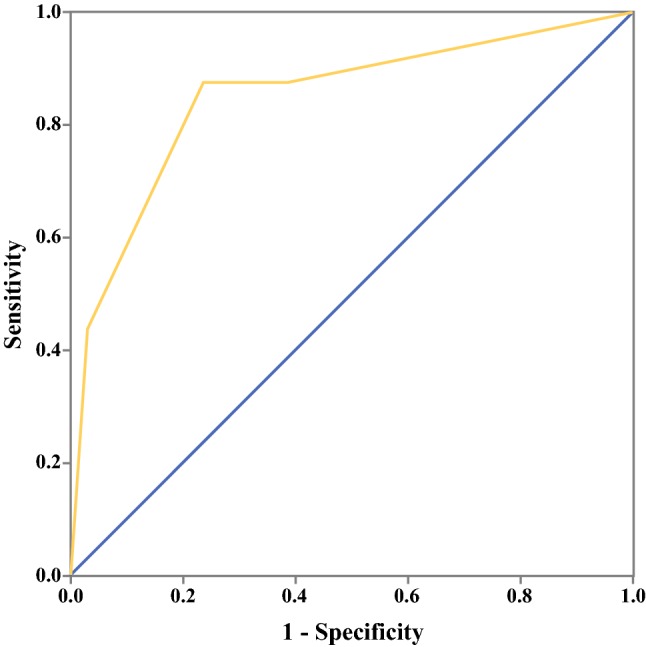
Receiver operating characteristic (ROC) curves to evaluate the diagnostic performance of the computed tomography (CT)-score indicator for prediction of staging laparoscopy results of occult peritoneal metastasis (OPM). The area under the curve is 0.848

This prospective study of 143 patients with gastric cancer showed a significant difference in OPM statuses among different CT score groups. An increase in CT scores was associated with a greater tendency for OPM positivity (Table 3; score 0, 2.5%; score 1, 0%; score 2, 21.2%; score 3, 63.6%; *p* < 0.001). The final scores were merged into two groups and subsequently compared with the findings of staging laparoscopy. A cutoff CT score of 2 or higher was associated with false-negative rate of 2% (2/99). The CT score system had a sensitivity of 87.5% and a specificity of 76.4% for OPM positivity (area under the curve [AUC], 0.848) (Fig. 3), and the true-positive rate was 31.8% (14/44). The agreement on the assigned final score was 76.2% (kappa, 0.5) between the two radiologists.

**Table 3:** Distribution of computed tomography (CT) scores for occult peritoneal metastasis (OPM)-negative and OPM-positive gastric cancers in a prospective cohort of patients

Score	OPM −	OPM +
0	78	2
1	19	0
2	26	7
3	4	7

*p* < 0.001, Spearman correlation analysis

## Discussion

In this study, staging laparoscopy was performed for 385 consecutive patients. The initial CT showed no definitive signs of peritoneal metastasis but further detected 33 patients with OPM. The most common OPM location missed by CT was the greater omentum, which also showed the highest CT score. This implies that abnormal signs were easier to detect on the greater omentum than at other sites.

For 22 patients in this study, OPM on the greater omentum was detected by staging laparoscopy. Only two patients had no abnormal CT signs. In the remaining 20 patients (90.9%), OPM-positive areas exhibited smudge opacity, displayed as patchy and very dense accompanied by strands, vague small nodules, or both. This sign was similar to the ground-glass opacity (GGO) observed in the lung, but in a fat-dense background. Therefore, we named it as smudge-like ground-glass opacity (S-GGO).

A previous study had proposed that the intraperitoneal free cancer cells specifically deposit in the lymphatic stomata and proliferate in the sub-mesothelial lymphatic space.^8^ This may cause proliferation and increased blood supply to the peritoneum, which may be the histopathologic basis of the S-GGO sign on CT. According to another study,^9^^,^^10^ carcinoma-associated fibroblasts (CAFs) drive peritoneal dissemination of gastric cancer and may contribute to the fabric strands displayed on CT.

The aforementioned theories are plausible explanations for the histopathologic basis of S-GGO. However, the exact histopathologic mechanism of the S-GGO sign needs to be studied further.

The parietal peritoneum was the second most common region for occult peritoneal metastasis, which can be detected by its contrast to the nearby low-density fat tissue. The normal parietal peritoneum usually displays as an ultra-thin membrane structure. The metastatic parietal peritoneum can appear to be uniform or to have irregular thickening, sometimes together with tiny, highly enhanced nodulars. However, most of the parietal OPM missed by CT in this cohort appeared as uniformly thickened line-like structures, which were likely to be missed due to their lack of nodularity.

The perihepatic peritoneum was a common location whose involvement was hard to detect even with retrospective CT examination. In this study, 13 perihepatic peritoneal areas were involved, but the average score of CT detectability was only 0.85, which was the lowest among all the OPM areas.

The perihepatic peritoneum is continuous with the parietal peritoneum, which should have similar CT signs. However, because the perihepatic peritoneum is located in the narrow gap between the liver and the diaphragm, the metastasis of this area usually appears as diffuse miliary or flaky nodules, or as large-range nodules with a flat shape, which is difficult to detect by CT. However, ascites around the liver may be an indirect sign of OPM in the perihepatic peritoneum.^11^,^12^

The presence of at least 50 mL of ascetic fluid was shown to be strongly indicative of PM. In addition, detection of perihepatic ascites on CT usually indicated the presence of at least 50 mL of ascitic fluid. Therefore, OPM should be highly suspected in such cases.

This region-to-region comparison study of CT with staging laparoscopy provided a comprehensive observation of the possible locations for PM. The lesions were most commonly located in the greater omentum and the parietal peritoneum. However, other uncommon sites should also be noted, such as the transverse mesocolon, the hepatogastric ligament, the falciform ligament, the perihepatic peritoneum, and the left subdiaphragmmatic area.

This study had some methodologic improvements. First, the combined application of three-plane images facilitates the detection and localization of OPM (e.g., the transverse mesocolon can be displayed more intuitively on the sagittal plane). Second, the wider window width/level (*W*_w/l_) can be used to highlight the tiny lesions in fatty spaces, and to facilitate the detection of the S-GGO sign as well as the thin parietal peritoneum. According to our experience, the *W*_w/l_ should be wide enough to demonstrate clearly the homogeneous granular background of fat tissue and internal membranes and the small vascular structures.

We also performed a prospective verification in a cohort of consecutive patients. The results showed that the probability of OPM increased with the increase in CT scores. Only 2% of OPM-positive patients were missed if a CT score lower than 2 was used as a threshold to diagnose PM negativity before staging laparoscopy. In addition, approximately 31.8% of OPM-positive patients were further detected using a CT score of 2 or higher as the cutoff value.

These findings have important clinical implications. Staging laparoscopy may be unnecessary if the CT score is lower than 2, considering the low rate of OPM occurrence (2%). Considering that the inclusion criteria specified advanced gastric cancer (≥ cT2M0) as an indication for staging laparoscopy in the National Comprehensive Cancer Network (NCCN) guidelines,^13^ all 143 patients in the prospective cohort had this indication, whereas only 11.2% (16/143) had peritoneal metastasis during laparoscopy. When the CT score was used for patient selection, the need for staging laparoscopy could be obviated for 69.2% (99/143) of the prospective cohort. This scoring system provided us with a Breast Imaging Reporting and Data System (BI-RADS)-like system^14^ for further evaluation of the likelihood for peritoneal metastasis. In contrast, detection of a heterogeneous increase in fat density with patchy or intensive S-GGO on the greater omentum or obvious thickening of the line with enhancement of the peritoneum-covered organs or tissues on CT is a strong indication for staging laparoscopy.

This study had some potential limitations. First, although we referred to nearby organs and blood vessels to locate the OPM areas, it was difficult to perform a precise region-to-region match between two-dimensional CT images and three-dimensional laparoscopy views. Second, because most signs of OPM missed on CT were not very obvious (i.e., slightly increased S-GGO and thin membranes), it was hard to define these signs precisely with an objective, quantitative description. Radiomics and texture analysis may provide promising surrogate parameters for a more objective description.^15^ Third, because the scoring system was established using region-to-region comparison, it was theoretically impossible to include cytology status, and because the status of cytology could be obtained using bedside abdominal puncture preoperatively,^16^ the status of the cytology was not included in the results.

In conclusion, we explored the CT signs of occult peritoneal metastasis by performing a region-to-region comparison with findings of staging laparoscopy. Detection of a heterogeneous increase in fat density with patchy or intensive S-GGO lesions on the greater omentum or an obviously thickened line with enhancement of peritoneum is strongly suggestive of OPM. A 4-point score system was developed accordingly, and staging laparoscopy might not be necessary for patients with a score lower than 2.

## Electronic supplementary material

Below is the link to the electronic supplementary material.

Supplementary material 1 (DOCX 7145 kb)
