# Supplementary material for: Four-Point Computed Tomography Scores for Evaluation of Occult Peritoneal Metastasis in Patients with Gastric Cancer: A Region-to-Region Comparison with Staging Laparoscopy
Source: Ann Surg Oncol. 2020 Jan 21;27(4):1103–9. doi: 10.1245/s10434-019-07812-y (PMC7060153; doi:10.1245/s10434-019-07812-y)
Supplement: Supplementary file 1 — Supplementary material 1 (DOCX 7145 kb) [file 10434_2019_7812_MOESM1_ESM.docx]

**Table S1. The CT grades of 57 regions of OPM positive areas.**

| Patient number | Lesion number | Regions | CT grades |
| --- | --- | --- | --- |
| P1 | L1 | Falciform ligament | 2 |
|  | L2 | Perihepatic peritonum | 1 |
|  | L3 | Greater omentum | 2 |
| P2 | L1 | Perihepatic peritonum | 2 |
|  | L2 | Greater omentum | 2 |
| P3 | L1 | Greater omentum | 3 |
|  | L2 | Parietal peritoneum | 3 |
| P4 | L1 | Parietal peritoneum | 1 |
| P5 | L1 | Greater omentum | 3 |
|  | L2 | Parietal peritoneum | 2 |
| P6 | L1 | Perihepatic peritonum | 0 |
|  | L2 | Greater omentum | 3 |
| P7 | L1 | Parietal peritoneum | 2 |
|  | L2 | Perihepatic peritonum | 0 |
| P8 | L1 | Greater omentum | 2 |
| P9 | L1 | Parietal peritoneum | 0 |
| P10 | L1 | Perihepatic peritonum | 0 |
|  | L2 | Greater omentum | 3 |
| P11 | L1 | Parietal peritoneum | 2 |
|  | L2 | Falciform ligament | 2 |
|  | L3 | Perihepatic peritonum | 2 |
|  | L4 | Greater omentum | 3 |
| P12 | L1 | Greater omentum | 3 |
|  | L2 | Transverse mesocolon | 3 |
| P13 | L1 | Perihepatic peritonum | 0 |
|  | L2 | Parietal peritoneum | 2 |
| P14 | L1 | Parietal peritoneum | 2 |
| P15 | L1 | Perihepatic peritonum | 0 |
| P16 | L1 | Perihepatic peritonum | 0 |
| P17 | L1 | Parietal peritoneum | 1 |
|  | L2 | Greater omentum | 1 |
| P18 | L1 | Greater omentum | 2 |
|  | L2 | Perihepatic peritonum | 0 |
| P19 | L1 | Perihepatic peritonum | 0 |
| P20 | L1 | Left subdiaphragm area | 1 |
|  | L2 | Greater omentum | 0 |
| P21 | L1 | Greater omentum | 0 |
| P22 | L1 | Greater omentum | 3 |
| P23 | L1 | Greater omentum | 1 |
| P24 | L1 | Greater omentum | 2 |
|  | L2 | Parietal peritoneum | 2 |
| P25 | L1 | Greater omentum | 3 |
| P26 | L1 | Hepatogastric ligament | 3 |
|  | L2 | Greater omentum | 2 |
| P27 | L1 | Greater omentum | 2 |
| P28 | L1 | Greater omentum | 2 |
|  | L2 | Parietal peritoneum | 0 |
|  | L3 | Left subdiaphragm area | 3 |
| P29 | L1 | Parietal peritoneum | 3 |
|  | L2 | Perihepatic peritonum | 3 |
| P30 | L1 | Hepatogastric ligament | 2 |
|  | L2 | Transverse mesocolon | 2 |
| P31 | L1 | Greater omentum | 2 |
| P32 | L1 | Left subdiaphragm area | 0 |
|  | L2 | Parietal peritoneum | 2 |
| P33 | L1 | Greater omentum | 3 |
|  | L2 | Perihepatic peritonum | 3 |


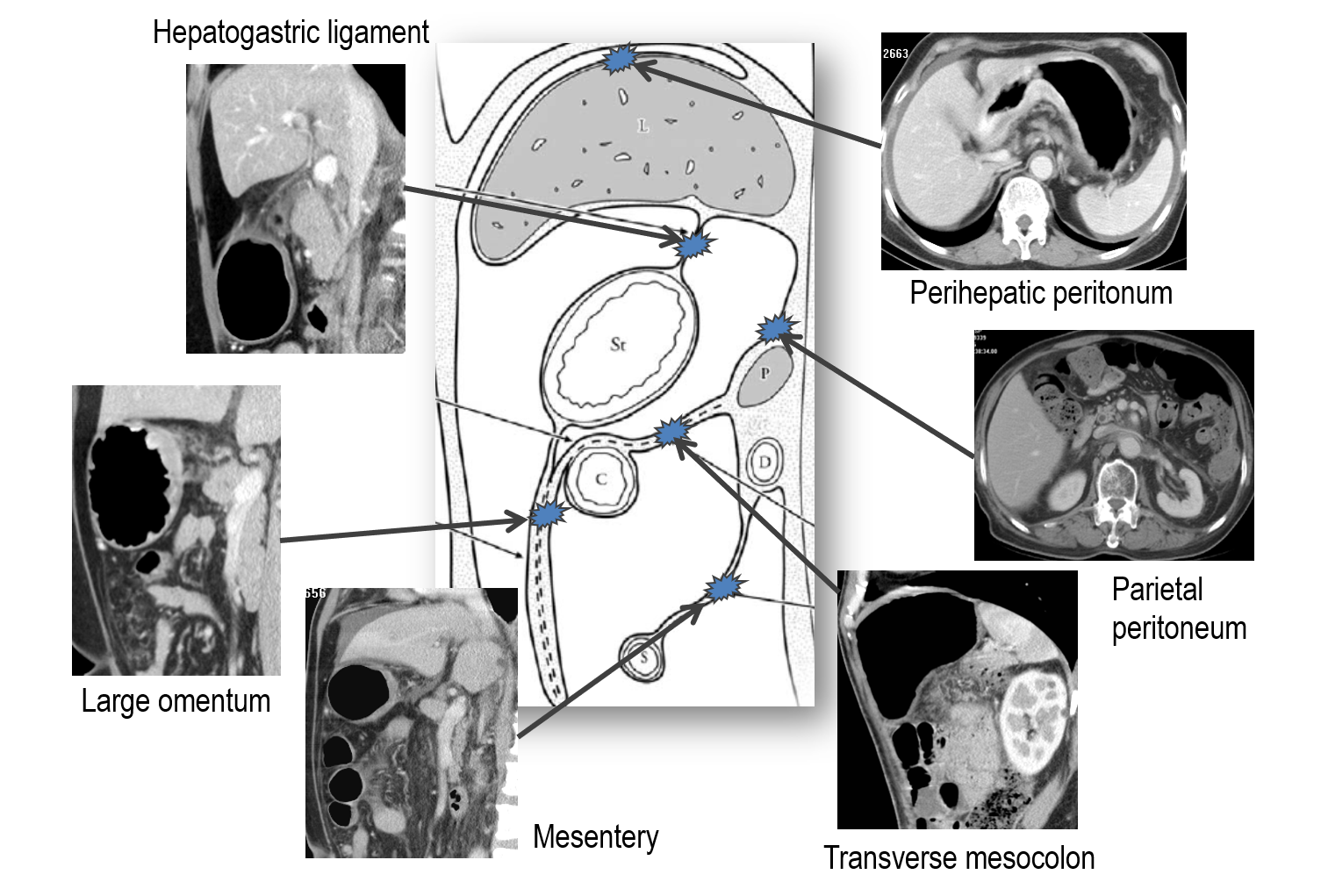


**Fig. S1. Anatomic locations of the PM lesions on CT compared with sagittal plane of pattern graph**

**Anatomic locations and reconstruction planes**

(1) Greater omentum: the fat density area extends from greater curvature of the stomach to the lateral and infra-anterior side of the colon, covering the hepatic and splenic flexures of colon and the ascending and descending colon. The axial and sagittal planes should be combined during observation.

(2) Transverse mesocolon: fat space between the transverse colon and pancreas, mainly observed in the sagittal plane.

(3) Perihepatic peritoneum: the peritoneum around the liver, observed in axial, coronal and sagittal planes.

(4) Left sub-diaphragm area: the free area under the left diaphragm, observed in coronal and sagittal planes.

(5) Parietal peritoneum: the thin-membrane structure between the fat of the inner-side abdominal wall and abdominal cavity, observed in axial and coronal planes.

(6) Mesentery: the fat density region along the mesenteric vessels, observed in axial plane.

(7) Falciform ligament: the hepatic fissure where the falciform ligament extended, observed in axial, coronal, and sagittal planes.

(8) Hepatogastric ligament: the fat space along the hepatic portal to the lesser curvature of the stomach, observed in axial, coronal and sagittal planes.


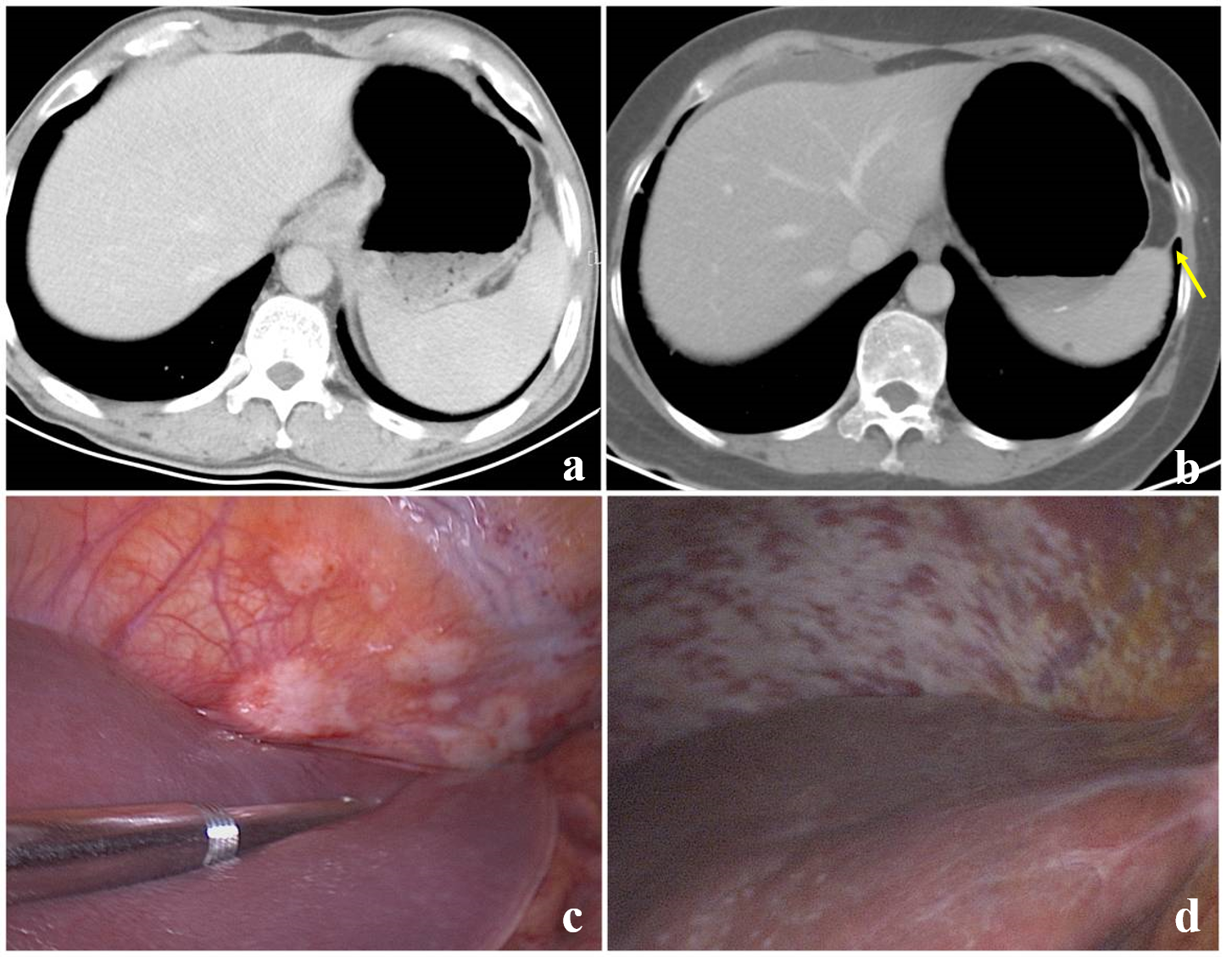


**Fig. S2. OPM on perihepatic peritoneum.**

a) Perihepatic peritoneum exhibiting no obvious thickening on CT; however, large flat-shaped metastatic lesions were observed by staging laparoscopy (c). b) perihepatic peritoneum exhibiting no obvious thickening; however, small amount of ascitic fluid was detected on CT (arrow); staging laparoscopy demonstrated diffusely distributed flat-shaped metastatic lesions (d). In this case, findings of ascites on CT played a key role in diagnosis.

OPM, occult peritoneal metastasis


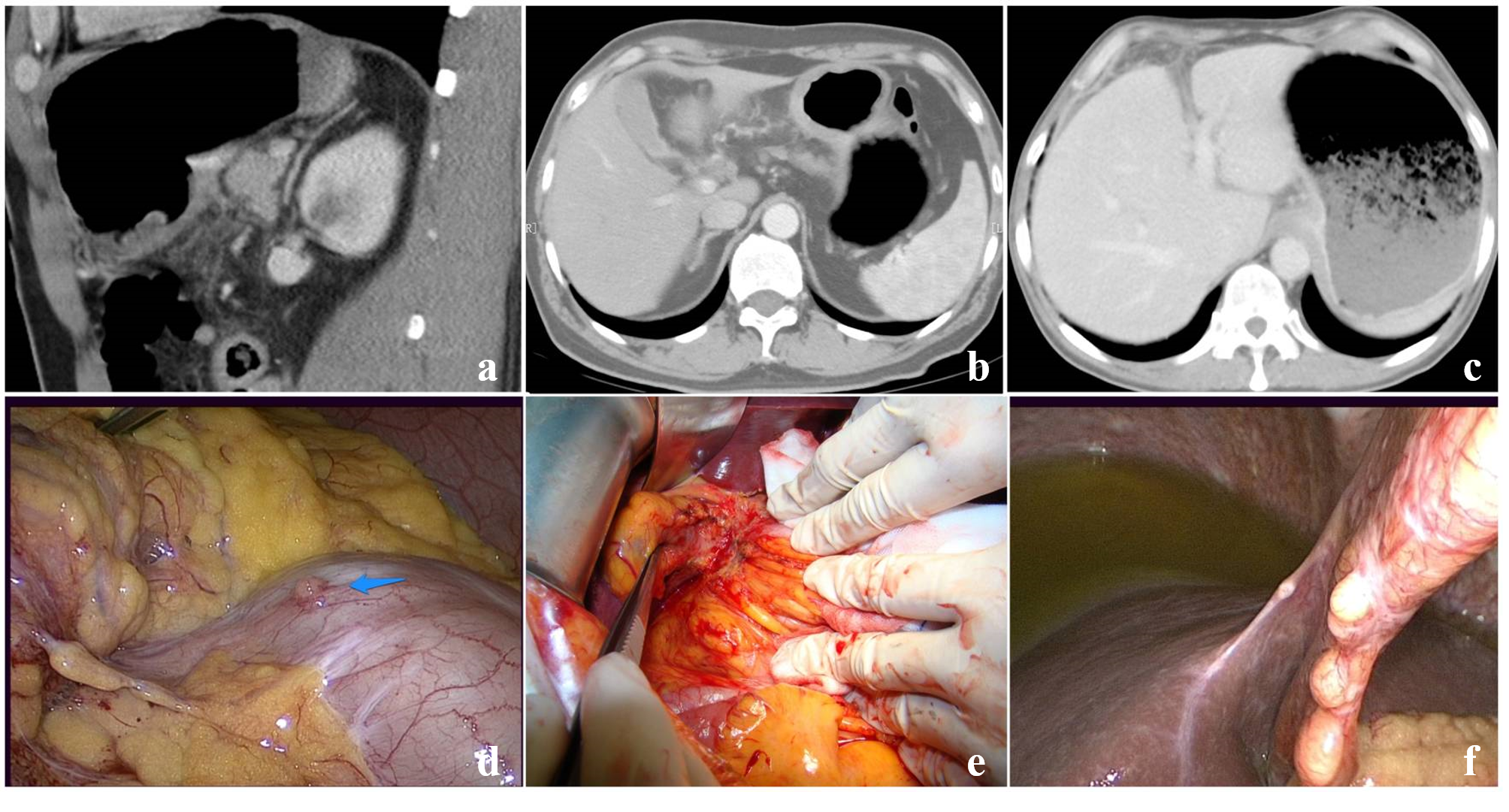


**Fig. S3. Uncommon OPM areas.**

a) Metastasis of transverse mesocolon: S-GGO sign was observed with multiple strands and blurred small nodules in the area between the pancreas and transverse colon (OPM grade 3). b) Metastasis of hepatogastric ligament (open-exploration): increased fat density with patchy S-GGO was observed in the area between the lesser curvature of the stomach and the hepatic portal (OPM grade 2). c) Metastasis of falciform ligament: S-GGO sign was observed with multiple strands (OPM grade 3). d–f) intraoperative screenshots of staging laparoscopy of the areas corresponding to the above CT findings.

OPM, occult peritoneal metastasis; S-GGO, smudge-like ground glass opacity

**Detail of CT examination**

The enrolled patients underwent abdominal CT (Discovery CT750 HD scanner, GE Medical Systems, Milwaukee, WI, USA) examination following overnight fasting. In the absence of any contraindications (glaucoma, prostate hypertrophy, asthma, or severe heart disease), 20 mg anisodamine was administered intramuscularly 15–20 min prior to CT examination to reduce gastrointestinal motility. All patients received effervescent granules (6g) to distend the stomach right before CT scan.

All patients underwent unenhanced and two-phase enhanced CT examinations (arterial phase: 40 seconds after injection; venous phase: 70 seconds after injection). All scans included the whole abdominal region. Non-ionic contrast material (300 mg/mL iohexol at a dose of 1.5 mL/kg body weight, 80–120 mL total; Omnipaque; GE Healthcare) was injected into the antecubital vein at the rate of 3.5 mL/s using an automatic power injector.

Imaging parameters of CT examinations were: spectral imaging mode with fast tube voltage switching between 80 kVp and 140 kVp during a single rotation; tube current: 640 mA; 5 mm collimation thickness; 0.625 mm reconstruction thickness; 0.6 s rotation speed; 0.984 helical pitch; and 22.82 mGy CT dose index volume. Patients were instructed to suspend respiration during scanning.

CT images were reconstructed using software designed to decompose projection-based images. An adaptive statistical iterative reconstruction (ASIR, index = 30%) algorithm was applied to suppress image noise and decrease the radiation dose.

The diagnosis was performed with a standardized dynamic window adjustment procedure on PACS workstations. First, a narrow window width/level (W_w/l_) was used to locate the primary lesions, and then the W_w/l_ was adjusted wide enough to observe the peritoneal fat status, which included all regions of the peritoneum and omentum. The optimal window for the detection of PM should clearly display mild grainy background noise of the fat tissue. Second, three-plane images (axial, coronal and sagittal planes) were observed to facilitate the detection of PM.
